# When smartphones take over: a mixed methods study of phubbing in child and adolescent psychiatry

**DOI:** 10.1186/s13034-025-00950-0

**Published:** 2025-08-22

**Authors:** Ahmet Büber, Brian Lu, Bürge Kabukçu Başay, Andrés Martin

**Affiliations:** 1 Pamukkale University, Pamukkale, Denizli 20160 Türkiye; 2 Yale Child Study Center, 230 South Frontage Road, New Haven, CT 06520-7900 USA

**Keywords:** Phubbing, Digital health, Child and adolescent psychiatry, Qualitative methods

## Abstract

**Introduction:**

The recent term *phubbing* is the amalgamation of the words *ph*one and sn*ubbing*, and refers to those phone-related behaviors through which we ignore, dismiss, or otherwise eschew social interactions. Little is known about phubbing among child and adolescent psychiatrists (CAPs), a group often called upon to provide direction on how to guide children in their rapidly evolving cybernetic contexts.

**Methods:**

We conducted a mixed methods study of trainees in CAP (*n* = 73; 68% women), recruited in the US (6 training programs; *n* = 35) and Türkiye (5 programs; *n* = 38). For the quantitative component, we administered two standardized tests: the *Generic Scale of Phubbing (GSP)*, and the *Generic Scale of Being Phubbed (GSBP).* For the qualitative component, we conducted site-specific focus groups. After transcription, translation, and anonymization of the digitally recorded sessions, we analyzed the data using thematic analysis informed by interpretative phenomenology.

**Results:**

Younger participants scored higher on the GSP (*r* = -0.43, < 0.001), but ratings did not differ between countries (F = 0.65, df = 1, 70, *p* = 0.42). GSBP scores did not differ across age or country (*p* > 0.05). Through thematic analysis we arrived at a four-domain model: (1) *Perceptions*: regarding the role of smartphone use in modern society and their social implications); (2) *Explanations*: respondents’ conceptualization of antecedents to phubbing behaviors; (3) *Consequences*: specific outcomes, such as normalization or split attention; and (4) *Recommendations*: strategies to address phubbing and problematic phone use.

**Conclusions:**

Phubbing is a ubiquitous behavior that can have social and emotional consequences. Through a more nuanced understanding of their own phubbing practices, CAPs can modify maladaptive behaviors of their own, have a more empathetic understanding of phubbing by youths under their care, and provide more realistic guidance regarding smartphone use to patients and their families.

**Supplementary Information:**

The online version contains supplementary material available at 10.1186/s13034-025-00950-0.

## Introduction

Electronic devices and smartphones have become an essential part of modern life, serving multiple purposes in education, business, entertainment, and communication. The use of smartphones has specifically increased in recent years, with usage reaching approximately 70% of the global population by 2023 [1]. This trend is pronounced among children and teenagers as well: 31% of 8-year-old children [2], and 95% of teenagers in the United States have a smartphone [3]. Smartphones make life easier in many ways, but they can also cause various problems, including the potential impact on mental health among children and adolescents: problematic smartphone use has been linked to increased likelihood of anxiety, depression, and stress in children and adolescents [4]. These devices can also negatively affect sleep quality and duration in adolescents, impacting their performance and well-being during the day [5]. Further, they can have distracting effects and a negative impact on memory [6].

The impact of smartphones extends beyond mental health, changing the very nature of human interactions. This shift has introduced a new phenomenon into our lives: *phubbing.* Phubbing, derived from the words *ph*one and sn*ubbing*, can be defined as the behavior of interacting with one’s phone at the expense of ignored others around oneself [7]. There are few studies on the prevalence of phubbing, but a study conducted among young people aged 15–29 showed a prevalence of 49% [8]. Another study conducted in Spain among young people aged 12–21 found that 17% of participants reported phone distraction during social interactions [9]. Phubbing is common among young people, and an Australian study has confirmed that young individuals exhibit the behavior more often than older people [10].

Research has shown that phubbing is especially directed toward people who are close, rather than more distant ones [10]. Phubbing can negatively affect the quality of communication: [11] its impact spans across friendships [12], parent-child relationships [13], work relationships [14], and romantic partnerships [15]. Being phubbed by others might lead to lower relationship satisfaction, greater disappointment [15], and depressive symptoms [16]. Antecedents to phubbing have also been studied and include: loneliness, fear of missing out (FoMO), boredom, and multitasking [17]. Additionally, a systematic meta-analytic review examined the predictors of phubbing behavior and showed a strong association between problematic smartphone use with phubbing, and a moderate association with psychopathology and being phubbed by others [18].

Considering the results of phubbing and its close relation with problematic smartphone use, this phenomenon is understandably of concern to parents, educators, health practitioners, and policymakers. And yet, research on phubbing struggles to keep pace with the uptake and evolution of the practice [19]. Despite recommendations from professional organizations about screen use in children and adolescents, specific guidelines for smartphone use remain inadequate [20, 21], leaving concerned individuals with limited access to reliable information.

Child and adolescent psychiatrists (CAPs) are regularly confronted with needing to provide guidance regarding electronic device and smartphone use among youth. However, they often have to rely on limited empirical information. In an effort to address this gap, we conducted a study through which to better understand the phubbing behaviors of CAPs themselves – both in their professional and their personal capacities. To our knowledge, no study about phubbing in CAP has been published before; our study’s unique premise is that that phubbing is a near-universal phenomenon that involves not only others (such as patients) but ourselves as well. In this study, we consider CAPs as both study subjects and potential change agents. We posit that insights from looking at our own practices can inform how we provide guidance to those under our care.

## Methods

We conducted a mixed methods study to explore the perceptions of trainees in child and adolescent psychiatry (CAP) about their personal use (and misuse) of smartphones, and of their professional role providing guidance on smartphone use to their patients and their caregivers.

### Participants and ethics approval

Through convenience and snowball sampling, we recruited CAP trainees (*n* = 73) from 11 programs and two countries: the US (*n* = 35; 6 programs) and Türkiye (*n* = 38; 5 programs). We conducted hour-long interviews with each program, using videoconferencing and digital recording with Zoom (San Jose, CA). Each of the 11 sessions consisted of a focus group facilitated in English or Turkish and loosely following a previously designed interview guide of sensitizing questions (**Appendix 1**). Focus groups had a median of 7 participants each (range, 2–10). In the last 5 min of the allotted hour, participants completed demographic and quantitative measures that they accessed on their phones by scanning a QR code linked securely to Qualtrics (Seattle, WA).

We obtained institutional review approval from the Yale Human Investigations Committee (Protocol # 2000039371) and the Pamukkale University Non-Interventional Clinical Research Ethics Board (Protocol # E-60116787-020-643288) prior to starting data collection. The study was deemed of minimal risk and exempted from full review. Participants were provided with study information and informed consent documents in advance of their session, at the outset of which they provided verbal consent, which was recorded. Participants agreed to maintain confidentiality within the group, and were assured that all digital recordings would be destroyed as soon as they were transcribed and anonymized (usually within 24 h). Moreover, we assured participants all their responses would remain confidential, and that neither their willingness to participate nor their specific responses would have any bearing on their training evaluations.

### Quantitative component

All participants completed a demographic survey and 2 standardized instruments:

*Generic Scale of Phubbing (GSP)*: The scale measures phubbing behavior in social interactions. It is a 15-item scale using a 7-point Likert scale (1 = never to 7 = always). The scale includes four factors: problem acknowledgment (PA); self-isolation (SI); interpersonal conflict (IC); and nomophobia (the fear of detachment from one’s mobile phone, NP). The total score is calculated as the average of all items. Higher scores reflect more frequent phubbing behavior. The GSP is highly reliable and has a Cronbach α of 0.93 [22]. It has been adapted into Turkish [23].

*Generic Scale of Being Phubbed (GSBP)*: The scale measures experiences of being phubbed in social interactions. It consists of 22 items using a 7-point Likert scale (1 = never to 7 = always). It includes 3 factors: interpersonal conflict (IC); feeling ignored (FI); and perceived norms (PN). The total score is obtained by averaging scores across all items. Higher scores indicate a greater experience of being phubbed. The GSBP is highly reliable and has a Cronbach α of 0.96 [22]. It has been adapted into Turkish [23].

We first calculated scale and subscale scores for the GSP and GSBP, and confirmed normality of variable distribution using the Kolmogorov-Smirnov and Shapiro-Wilk tests. We then used linear regression to compare scores while accounting for the effects of country and participant age. We dichotomized age into two birth cohorts: Millennial [1981–1996] or Generation Z [1997–2012]. We performed statistical analyses with SPSS 25.0 (Armonk, NY).

### Qualitative component

We used qualitative methods to learn about phubbing practices in the participants’ lives. We opted for small focus groups rather than individual interviews in order to foster rich discussion. The questions that guided this part of the study centered both on the *personal* relationship between CAPs and their smartphones (e.g., at home, at work, during leisure time), as well as when in their professional roles (e.g., the guidance they provide about smartphone use to patients, families, caregivers, or stakeholders).

We transcribed digital recordings in English or Turkish using DeepGram (San Francisco, CA). We translated Turkish transcripts by first using DeepL (Cologne, Germany), then ensuring accuracy through manual verification by a native speaker (AB). We next analyzed transcripts using thematic analysis (TA) [24, 25] informed by descriptive phenomenology (DP), which foregrounds participants’ experiences to identify the structure that is the phenomenon, rather than its personal interpretation by the researcher [26, 27]. TA provides theoretical freedom and flexibility to identify commonalities, and where writing and analyzing data can occur recursively alongside one another. Two authors worked independently to identify and compare codes before sharing them with the third investigators for triangulation [28], further refinement, and finalization into a streamlined codebook and overarching domains, themes, and subthemes. Each key theme was supported by multiple quotes. In keeping with the tenets of participatory research [29, 30], we consider our subjects to be co-investigators, and invited all participants to review and comment on our final codes and conclusions. We followed an inductive approach in which research questions evolved beyond sensitizing questions to explore new or unanticipated themes. We analyzed transcripts iteratively until we reached theoretical saturation [31] and followed best practice guidelines for the analysis, drafting, and submission of qualitative studies [32].

### Reflexivity and positionality statement

TA includes a rich and detailed account of the data and welcomes attention to the investigators’ reflexivity, the ability to examine one’s own influence on the research process [33]. The four authors were keenly aware of their own instances of phubbing, of having been phubbed, and of the challenges in addressing phubbing-related behavioral change in themselves and others. They approached the subject matter as one germane to their everyday lives, rather than as something happening exclusively to others. This perspective, complemented by their ability to conduct focus groups in the participants’ native languages, allowed them to have an “empathic foothold” on the phenomena under study. The authors did not have a supervisory or evaluative role on the subjects they interviewed, thus minimizing biases of power dynamics or social desirability.

## Results

### Quantitative component

The Turkish and American groups were comparable in gender composition (consisting of 76% and 60% women, respectively; $\:\chi\:$ ^2^ = 2.24, *p* = 0.13). Turkish participants were significantly younger than their American counterparts (29 ± 2 vs. 34 ± 3 years old, respectively; t = 6.83, *p* < 0.001); 18 of the 38 (47%) Turkish participants belonged to the younger Generation Z cohort, compared to none among the 35 American participants. GSP overall score was inversely correlated with age (*r* = -0.43, < 0.001). There was a significant difference in GSP between the Gen Z and Millennial cohorts (61.4 ± 10.9 vs. 52.7 ± 10.5; t = 17.54, *p* < 0.001). An apparent difference in GSP between the Turkish and American groups (59.4 ± 11.2 vs. 49.9 ± 8.8; t = 4.01, *p* < 0.001) dissipated after adjusting for age, by conducting a one-way analysis of variance with GSP as dependent variable, country as independent variable, and age as covariate (F = 0.65, df = 1, 70, *p* = 0.42). GSBP overall scores did not differ between countries or across ages (*p* > 0.05). Table 1 summarizes total and subscale scores for GSP and GSBP for the sample of 72 (one of the 73 participants did not complete the quantitative component). In response to the question “How many hours can you go during a typical day [excluding sleep] without engaging with your smartphone?”, participants averaged 3.6 ± 2.3 h.

**Table 1:** GSP, GSBP, and factor scores

	*n* = 72	
	Mean	StandardDeviation
**GSP total**	54.8	11.2
**Factors**		
NP = Nomophobia (fear of detachment from one’s mobile phone)	17.8	4.7
PA = Problem Acknowledgement	13.2	3.2
SI = Self-isolation	13.0	4.3
IC = Interpersonal Conflict	10.8	4.2
**GSBP total**	83.2	14.6
**Factors**		
PN = Perceived Norms	40.5	7.3
FI = Feeling Ignored	28.1	6.1
IC = Interpersonal Conflict	14.6	5.7
Note: GSP = General Scale of Phubbing; GSBP = General Scale of Being Phubbed.		

### Qualitative component

Through thematic analysis, we arrived at a four-domain model through which to approach phubbing in child and adolescent mental health: (1) Perceptions; (2) Explanations; (3) Consequences; and (4) Solutions. We provide brief explanations of these domains and their underlying themes in Table 2. In the sections that follow, we further explore each of the domains and themes. Consentient with our quantitative findings, we did not find notable differences between the US and Türkiye groups. Still, we go on to indicate attribution of specific quotes using the U or T suffix, respectively.

**Table 2:** Phubbing framework: domains, themes, and definitions

Domain	Theme	Definition
**Perceptions**		Roles of smartphone use in modern society and their social implications
	Active danger	Negative effects associated with smartphone use (e.g. bullying, echo chambers)
	Distraction	Neutral effects associated with smartphone use
	Legitimate tool	Positive effects associated with smartphone use (e.g. research, community)
**Explanations**		Respondents’ conceptualizations of antecedents to phubbing behaviors
	Person-centered	The perception of phubbing behaviors as internally or externally manageable
	Principle-centered	How higher-order concepts (e.g. ethics, empathy) affect phubbing
	Emotion-centered	How specific emotions (FoMO, anxiety, boredom, etc.) affect phubbing
	Economic/ Utilitarian	How perceived utility and risk/benefit analyses influence phubbing decisions
**Consequences**		Specific outcomes of phubbing behavior (e.g. normalization, split attention)
	Feelings	Emotional effects of phubbing on phubbers and phubbees
	Attention span and multitasking	Effects on attention span and split attention
	Socialization	Further social effects following the rise of phubbing (e.g. normalization)
	Clinical	Effects of phubbing in the clinical environment
**Recommendations**		Strategies to address phubbing and phone use trends as suggested by CAP fellows
	Incremental	Quantitative strategies to reduce exposure of children and adolescents to devices
	Categorical	Qualitative strategies to adjust how children and adolescents are exposed to and use devices

### Domain 1. Perceptions: smartphones and technology in a social context

This first domain concerns the connotations with which smartphone use and technology are perceived by members of society. Our respondents frequently discussed the use of smartphones for work, communication, searching on the Internet, leisure, and more — and at every conceivable waking hour of the day. Broadly, we found that these devices can be viewed as *active dangers* (negative connotations), neutral *distractions*, or *legitimate tools* (positive connotations). Because these ideas alter the lens through which phubbing is perceived. We first render explicit this underlying social construct in order to facilitate analysis (Table 3).

**Table 3:** Domain 1. Perceptions: smartphones and technology in a social context

Theme	Subtheme	Representative quotation
Active danger	Bullying	A lot of the chief complaints have to do with bullying and problems that happened on their phone, whether it’s social media or text, or whatever platform between their friend groups. (01U)
	Exposure to polarizing content or misinformation	It can be very dangerous, skewing their worldviews in certain ways. (06U)
	Hindrance to social development	It’s very much related to regression in young children…For primary school children, it’s usually related to a decline in academic success, but for adolescents, it can lead to risky behaviors like alcohol, substance use, or abusive relationships. (07T)
Distraction		I might miss something that’s said, or when I’m saying something to the other person, they might miss it. It creates more problems in the work environment, not so much in the friend environment (07T)
Legitimate tool	Research and work	It will aid in the discussion, that we’re having an informed debate about something (12U) The phubbing will at times contribute to this patient’s care; it is happening in real time; it can enrich the conversation and imbue it with facts. (01U)
	Patient connection	I think it’s complicated because, in therapy, you have to do work and you don’t let someone on your phone, but, also, my kids want to show me things. And it helps move them on to the next topic without looking at it so closely. And so, I allow it to a certain point: helpful for rapport and for feeling heard. (06U)
	Connection to community	In the course of two or three hours, my husband is at work trying to get a hold of me. My parents are somewhere else in the country. They need to connect with me. (01U)
	Personal health	Because I have diabetes, I use a sensor. I need to constantly open and check the sensor. So, I don’t have the luxury of staying away from the phone. It’s always in my pocket or by my bedside when I’m sleeping. It beeps when it falls, so I need to hear it. (07T)

#### Active danger

Responses we obtained coalesced around three key subthemes: *bullying*, *exposure to polarizing content or misinformation*, and *hindrance to social development*. Participants were concerned with phones and similar devices opening a doorway to increased dangerous interactions online, with cyberbullying being especially relevant for children and adolescents. Similarly, discussions of the danger of exposure to polarizing content or misinformation often centered on the ways in which online content may introduce naive phone users to trends and communities which shift normative viewpoints towards more fringe perspectives:

The echo chamber that happens in a lot of places like Reddit, for example, where you’ll have views that aren’t extreme in a lot of ways, and people that gravitate towards those views end up repeating the same and become more extreme in a way. (02U)

Other concerns included children and adolescents’ potential encounters with age-inappropriate content online and how such encounters may interfere with the usual sequence of social development:

I’ve seen that they become more introverted and develop more social anxiety symptoms. I also think that because they can easily access everything, they encounter sexuality too early, and dark things such as drugs, substances, alcohol (04T).

#### Distraction

Phones were regarded in a slightly more neutral manner when discussed as mere distractions, providing little or no value to the user, but at least not functioning as an actively harmful exposure. Most commonly, this perception of phones arose in the context of educational settings or didactics:

Sometimes training hours become like a time slot specially crafted for looking at phones. Yes: disengaging during training hours may be one of the times we do it most. (10T)

#### Legitimate tool

The previously described associations were occasionally contrasted with specific instances where phone use was considered legitimate and justified. For instance, the purposeful use of phones for research and work to augment productivity and learning was generally regarded positively:

I think of an attending telling us the story about how he used to ban cell phones from…rounds and everything, and then people would get yelled at for it. And then he came to realize that what was happening is people are actually, in real time, looking up medication facts to share. (01U)

The judicious use of phones for patient connection was usually regarded similarly. Several CAP fellows recounted utilizing a young patient’s phone as a window through which to gather clinically relevant information about their interests, personality, habits, or online social networks. This connection-promoting capacity of smartphones extended to the participants’ personal lives, helping them maintain connections to community and family when in-person interaction may not be feasible:

I also have family that’s all over the country, you know, old ones, young ones, and people that are still looking to me to take care of them even though my husband and I are far away. (01U)

Lastly, phones were also described as promoters of personal health in their own right, often via the monitoring of health data that can be illuminating for patients and clinicians alike:

I’ve also started having them use a lot of the mood tracking apps, to help them build insight into their moods and states, and then we can look at it together. (02U)

### Domain 2. Explanations: antecedents to phubbing

A second domain characterizes antecedents to phubbing by dissecting the reported thought patterns, emotions, and justifications behind participants’ phubbing of others, or how to react when phubbed by others. We found that many of these factors promoted phubbing, yet some effectively dissuaded respondents. By examining these antecedents, we may generate further ideas for sustainable interventions to modulate young patients’ phone use. Our analysis led to four common themes, which we term *person-centered*, *principle-centered*, *emotion-centered*, and *economic or utilitarian frameworks*. (Table 4).

**Table 4:** Domain 2. Explanations: antecedents to phubbing

Theme	Subtheme	Representative quotation
Person-centered	Internal locus of control	I turn off notifications for a lot of things. So that it’s not popping up other than maybe for work stuff; for everything else, notifications are turned off. (12U)
	External locus of control	I started messaging someone else when my fiancé and I were out for a thing and I’m like, man, I go to stop being on my phone, and then, *thankfully*,* it died*. (01U)
Principle-centered	Responsibility to patients	It wouldn’t be appropriate if the patient makes an appointment and comes to see us. It’s time we’ve allocated for them. Looking at the phone then, rather than looking at the patient, it just wouldn’t be right. (10T)
	Respect for authority	I would definitely phub my very close friends, my brother, my wife, my parents. But I’m more careful around people I respect and communicate with, like fellows or professors. (04T)
	Empathy	I get annoyed when other people do it, so I think I have a fairly conscious effort in terms of not doing it. But if I do end up doing it, I like to think I catch myself. (03U)
Emotion-centered	FoMO	We have a system on our phone from work… If we get a message from there, you are prompted to look at it because something could be happening. Has something happened to my patient? You know, you cannot always ignore it. (01U)
	Social anxiety	When I feel socially anxious, I give myself to my phone, I retreat. (04T)
	Boredom	There isn’t just one trigger. In many cases, it can be simple boredom. Especially in a crowded environment, and when the topic being discussed might not interest me. (07T)
	Escapism	If I don’t want to talk at that moment, turning to my phone is an avoidant behavior (10T)
Economic/Utilitarian	Attention economy	I think there’s also a slight competitive drive to it, in the sense that, like, I know these companies are trying to win my attention, and so trying to defeat them a little bit on their turf, on their wily ways, becomes a bit of a game for me. (05U)
	Risk/benefit calculations	If I’ve decided that if a lecture, for example, is not engaging or high yield, I am more likely to tune out and may resort to my phone. If the speaker is engaging or is giving high yield information, I’m unlikely to do so because that might offend them. (06U)

#### Person-centered framework

The crux of the person-centered framework hinges on how respondents conceptualize the responsibility of managing their phone use: whether proactively and in control of their habits, or reactively, in response to external factors. Those who endorsed an internal locus of control often appeared comfortable triaging concurrent demands on their attention and exhibited a sense of self-awareness of potential distractors:

I like to put my phone on do not disturb a lot because nothing is really usually that urgent. (05U)

In contrast, those who endorsed an external locus of control more readily disavowed agency for controlling their phone use, instead attributing the blame to something other than their intentional selves:

It’s like a habit, not because I’m uncomfortable with what’s being talked about or not very interested, but in that empty moment, suddenly my hand goes to it. (07T)

At times, when feeling pulled to their devices, setting external barriers could prove the only plausible way to desist: turning the device off, giving it away for temporary safekeeping, letting its battery run out, or most commonly, relegating it to another physical space.

I put my phone in another room from where I’m at. (02U)

#### Principle-centered framework

Other times, respondents cited first principles such as ethics, respect, or empathy when presented with a dilemma involving phubbing and phone use. This top-down pattern of thinking assumed the necessity of a particular end goal (e.g., ethical responsibility to patients), and worked backwards to consider whether actions such as using one’s phone were precluded or permissible. For instance, a striking finding among the focus group responses was that CAP fellows almost never phubbed a patient:

It would be rude if I did it in front of my professor. You know, they might get angry. Doing it in front of a patient could be one of the worst things possible. It would probably directly damage the therapeutic relationship. (07T)

Compared to ethics, respect for authority was less universally espoused among respondents, but just as strongly adhered to among those who did. Frequently, this sense of respect was in deference to attending physicians or professors in the clinical/academic environment and parents or elders in personal life.

They’re imparting knowledge to us, dedicating their time to us. I mean, if we look at the phone during that time, I think we all feel we’re being disrespectful, and we think it might be perceived as if we’re not listening. (08T)

With regards to considerations of empathy between phubbers and phubbees, perceived similarities fostered a sense of forgiveness and mutual understanding:

No. I haven’t done anything outright to point them out or anything. You know? I’m not trying to be like a micromanager and because I do the same, I’m guilty of the same thing. (05U)

On the other hand, perceived dissimilarities in stature often led to the dissuasion of phubbing:

If the same was done to me, I would be very seriously disturbed and probably wouldn’t make an appointment at the same place again, or if it was a consultation, I would probably say I want to see another doctor. (07T)

I feel like there’s much less generous interpretations of phone use by older folks because I feel like their assumption is always that you are up to no good (06U).

#### Emotion-centered framework

Yet another pattern of thinking about phubbing revolved around the inciting emotions (e.g. *FoMO*, *social anxiety*, *boredom*, and *escapism*) which inclined respondents to phub others. For example, some CAP fellows endorsed a strong drive to be maximally updated on current events or trends, prompting increased device use and phubbing:

Am I missing something that’s happening? What happened in the country today? I feel like I need to check in. (08T)

This worry about not knowing presented itself in clinical contexts as well:

That messaging center thing that we have is still on. So, when that dings you are like, oh, what happened there? You know? Was there a crisis that happened? (01U)

Social anxiety and boredom each functioned similarly, inclining respondents toward phubbing. Still others reported feeling the occasional need to retreat to technology as a type of withdrawal defense:

When I’m in an environment or talking to someone, if I don’t want to be fully present there and want to escape from that environment, I worry about how I’ll escape if my phone isn’t with me. (09T)

Young patients can also exhibit similar behaviors, especially in the clinical environment when in-person interaction with a psychiatrist may present as a situation outside of their comfort zone: it may take gentle limit setting to make the therapeutic space a phub-free zone. Phubbing during therapy (a not unheard of occurrence) could be taken as an invitation to discuss not just what is being avoided in the therapy, but what is being sought in the ether.

#### Economic/utilitarian framework

This final framework, more pragmatic than the others, arises when one consciously makes comparisons of value, estimations of risks, and evaluations of what may be gained or lost by deciding to phub in a particular moment. Consistent with the concept of attention economy, CAP fellows occasionally analogize attention to currency — they are astutely cognizant of its limited quantity and the increasing array of concurrent attention demands from clinical notifications, personal communications, and more:

Both tasks are important, but since stronger stimuli come from the phone, I devote all my attention there and my selective attention is blocked. (04T)

Still others explicitly verbalized how they weigh the risks of phubbing against perceived benefits:

If I’m in a lecture and I don’t think it’s as important as some work I need to get done, I will go on my phone. (01U)

### Domain 3. Consequences: outcomes of phubbing

Our third domain explores the outcomes of phubbing behavior, ranging from the reflexive impacts on the phubber to broader societal impacts. Whether as participant, observer, or victim, many respondents reported firsthand experience with the consequences of phubbing. These shared experiences contribute to the reproduction of altered social norms of in-person interaction among many of our respondents and their young patients. We categorize these outcomes into several themes of *feelings*, *attention span and multi-tasking*, *socialization*, and *clinical consequences* (Table 5).

**Table 5:** Domain 3. Consequences: outcomes of phubbing

Theme	Subtheme	Representative quotation
Feelings		If I’m on my phone and somebody calls attention to it, I’m not angry, but I feel called out, and then I feel embarrassed or guilty, and it comes out as defensiveness, even when it’s my husband. (01U)
		I think it makes the other person feel worthless. It inevitably made the other person feel that. (04T)
Attention span and multitasking		TikTok can be such a time suck. It’s just constant scrolling for new information, and it really has affected people’s attention span and the need for a quick dopamine hit. (05U)
		TV and my phone. That’s what I’m doing. Can’t watch a show anymore without having another screen to scroll through. (02U)
		I’ve got literally 10 tabs open right now. I usually have a split screen with whatever it is I’m doing, on video and also whatever else I want to work on, and then I also have my phone. (06U)
Socialization		Everyone is scrolling on Instagram and having this half presence with everyone else around them. (03U)
		We lie on separate couches at home, despite being so close together, we’re just sharing Instagram Reels with each other. (11T)
Clinical consequences	Effect of phubbing on clinical care	I have a ton of adolescent patients…but some walk into my office and their parent hands them the phone, and then I can only talk to the parent…I’ve had parents try to take work calls during appointments as their kid is telling them about things like trauma. (01U)
	Wish for resources and guidelines	I think I want to look into resources that can help parents with that; structured ways, basically behavioral interventions, that we do to promote phone hygiene. (01U)
	Role modeling	I’m telling the kid, hey, could you put your phone away? But I’m also trying for the parent to do the same. And if the parent doesn’t listen, the patient’s not going to listen. (12U)
		I’m saying, ‘you should not be on your phone in bed,’ but know that I do it all the time. (03U)

#### Feelings

The phubber and phubbee may perceive the act through different social and emotional lenses, resulting in distinct impacts on each party. To phubbers, device use in the moment may bring a sense of utility and joy:

In my social life, I feel as if the communication I have on the phone makes me even happier at times than verbal communication…I feel as if I am getting a reward when the message notification sounds. (04T)

Conversely, being phubbed is often described in a strongly negative light:

I don’t want to keep talking badly about my home life, but that’s where phubbing usually happens. And I think that’s where I feel undervalued and almost lonely…it’s you and me in this house, and, obviously, there’s something more important going on for you…than me existing here. (01U)

#### Attention span and multitasking

Often reporting from personal experience, CAP fellows also recognized deleterious effects on attention span as another major consequence of phone use trends:

A person might look at their phone unconsciously too. Actually, independent from whether they like the topic or not, it’s happening. Maybe interest and engagement exist, but attention spans have momentarily shortened - I mean, they seem to have shortened for many of us. (11T)

Correspondingly, it is no surprise that respondents are observing an increased propensity to multi-task, including across more than one technological device:

He’s a 13 year old boy with ADHD…and he’s playing the game. And then over here, he’s watching some other kid playing a video game. Meanwhile, the kid playing the video game is listening to music. (02U)

#### Socialization

Some focus group participants described a concern as child psychiatrists about maladaptive replacement of in-person socialization with device use. In some cases, using technological devices in parallel can becomes a social act of sorts, an approximation:

I think that younger age groups play e-games together, for example sometimes while waiting in corridors all children are looking at their phones, they don’t communicate, they don’t actually play together. (10T)

Fellows themselves also report experiencing technology use in this manner and perceiving these behaviors as normalized, a new kind of parallel play:

My husband and I find us doing that…I’ve got my laptop on my lap, and I’m playing video games on the PlayStation. And then I find he’s doing the same thing on his big screen that’s right next to him. And then I’ll look over and we’re both on our phones with these other two activities. (01U)

#### Clinical consequences

The most pertinent consequences of phubbing and maladaptive phone use to child and adolescent psychiatry are the implications for the clinical care of patients. Our participants observed firsthand how phubbing in the clinical environment can be dangerous, including directly interfering with emergent psychiatric care:

I actually had a patient yesterday…he’s sitting in this psych pod in the ER, on Instagram. And, while we were interviewing him, and multiple redirections to get his attention…to the point of having to be really stern. Because he had his mom’s phone. Obviously, he’s not allowed to have phones and stuff in the ED, but he’s about maybe 12…there was obviously something on Instagram that was far more interesting. How do we compete? (12U)

Still, CAP fellows generally have ideas about next steps that would better prepare them to tackle this issue. Some are aware of reputable published guidelines about children’s phone use, but others seek more support and resources about how to effectively address these concerns in their clinics.

Nonetheless, ongoing challenges complicate the feasibility of developing, disseminating, and executing the needed guidelines. Many fellows admit their own shortcomings with controlling their own phone use habits, and reasonably exhibit skepticism about children’s adherence to any such guidelines:

If we can’t regulate ourselves as adults, we can’t expect the little ones that we’re seeing to regulate themselves. (01U)

Others worry about the ability of overwhelmed parents to effectively enforce phone use limitations, and still others worry that the opportunity for psychiatric intervention presents itself at a later age than would be optimal to prevent the initial crystallization of maladaptive phone use habits.

### Domain 4. Recommendations: strategies to address phubbing

Despite the aforementioned challenges, CAP fellows were able to offer various recommendations based upon their personal lives and clinical experience managing young patients’ phone use. We have divided these insights into *incremental* and *categorical* strategies (Table 6).

**Table 6:** Domain 4. Recommendations: strategies to address phubbing

Theme	Subtheme	Representative quotation
Incremental strategies	Detox or tapering	I’ve been sober from Instagram for fifteen months now. (06U)
		If they’re looking at the screen for very long periods, we say they can reduce it gradually, that they can’t zero it out all at once (08T)
	Replacement activities	We say it needs to be filled with things like sports, courses, or hobbies, making social connections. Otherwise, they’ll cling to the phone again, or there will definitely be tantrums, they’ll demand it. (08T)
		I’m seeing that in some of my patients they are purposefully disconnecting from their phones now, and they’re taking up things like cross stitch or painting, and they’re doing word searches, you know, in their free time or, getting back into reading (01U)
	Delayed age of introduction to technology	I think the AAP’s recommendation is wait till eighth. So, typically, parents should wait until their child is in the eighth grade, so, like, 12, 13. And I’ll make that suggestion. (06U)
	Sanctuary hours	I personally don’t, use the phone from six to 08:30 every day because that’s my kids’ special time, and I’m focused on the kids. From 08:30 to 09:30, yes, I’m on my phone, and it goes to the silent from 09:30 onwards. (01U)
Categorical strategies	Device limitations	Then what about a flip phone? And, like, I don’t know if they’re going do it or not, but the kid was not happy when I suggested that. (03U)
	Active parenting and monitoring	So sometimes I have to, like, remind the parent that they’re the parent too. So they may have to do so things differently. It’s even hard for the adults to control this. (03U)
		You can’t completely shut it off, they’ll be exposed to it from friends in their environment too, but at least let the screens you give be ones where you can control the content, know what they’re watching. (09T)
	Increasing buy-in from children, adolescents, and families	I think there’s a really good conversation that we can have with teens and adolescents from a motivational interviewing perspective and really helping them understand that the same way that we talk about substance use or smoking cigarettes back in the day; how these corporations spend a lot of money trying to force you to do this and you have to be individual and be free. (01U)
		I ask them to fill out a diary a bit more like to have the child track their screen times, to help them understand the magnitude of the problem at first. (10T)

#### Incremental strategies to address phubbing

These strategies aim to modulate the overall quantity of time during which children and adolescents are permitted to engage with technological devices. For instance, some fellows stated that on occasion, advocating for total “detox” from the phone and altogether removing the maladaptive stimulus could be a necessary step:

Maybe because of safety concerns and whatever else may be going on we do detox…we recommend monitored time, but at times we need to take the phones away, especially if it’s becoming a safety concern, and see how they do. (01U)

Others disagreed, preferring to promote a more gradual tapering strategy instead:

Some parents I’ve seen do pretty draconian things, like no phone for Google or, tracking apps on their kids’ phones. I agree that we can try to curb excessive use. And depending on your bedtime, we need to go to bed. But cutting off all private accesses, that will just backfire tremendously. (02U)

Participants also emphasized the need to support children and adolescents in pursuing non-digital activities to prevent backsliding into ingrained phone use habits, delay the age of their introduction to personal devices and online platforms, and carve out specific hours when phone use is forbidden with the hopes of modelling improved compartmentalization and time management skills.

#### Categorical strategies to address phubbing

Complementary to the incremental strategies are qualitative approaches that seek to adjust how children and adolescents engage with technological devices. These categorical strategies include *device limitations*, *active parenting and monitoring*, and *increasing buy-in from children*,* adolescents*,* and families*.

Providing adolescents with an inherently limited device capable of basic functionality but with fewer opportunities for distraction or unanticipated use, such as an older-style flip phone or wearable smartwatch, was occasionally suggested by the respondents. While potentially time consuming, parents could also reinforce more adaptive habits by actively engaging with opportunities to monitor children and adolescents’ technology use. One easy key action item was for parents to invest the time in fully acquainting themselves with the capabilities of the devices they intend to let their children use, and especially any ready-to-use parental safeguards:

I’m always surprised by how underutilized the parental controls are on phones. Parents don’t even seem to know about them or how to use them. Because I know, at least on iPhones, which I feel like a lot of kids have iPhones, they have pretty robust time controls. (05U)

A sense of investment from the entire family is needed to extend the benefits of psychiatric intervention beyond the short clinic visits. A maximally effective strategy should also move children and adolescents to begin practicing self-management techniques and encourage them to request parental or clinician help as needed. In one respondent’s words:

Parents can block these things, but they will outsmart their parents and then figure it out. So I think instead of just blocking, having conversations, more than once. And then ask them, the teenagers…What would be the best way?…And then come up with realistic solutions, together. (01U)

## Discussion

At a time when reliance on phone connectivity is ubiquitous, and when children and adolescents have access to smartphones at ever younger ages, phubbing has become a public health and policy concern. All of our participants acknowledged phubbing and being phubbed as routine occurrences in their daily lives. However, the self-reported average of almost four hours that participants claimed to be able to go without using their smartphone is a likely underestimate, whether due to underreporting, social desirability, or unrealistic perceptions. For example, current estimates suggest that cell phone users look at their phones an average of 144 times a day [34], and underestimates of personal use can vary as much as 40% different from objective measures [35]. The fact that nomophobia (the fear of detachment from one’s phone) was the highest-rated subscale on the GSP further supports just how reliant participants were on their phones, even if not fully aware of the automatic reliance on their devices.

Cultural factors may impact phubbing and related perceptions. For example, in a large study (*n* = 14,700) in which participants rated vignettes related to the behavior, members from “collectivist” countries scored higher on phubbing scales than “individualist” ones. Moreover, “attribution locus” differed notably, with “individuals from collectivist backgrounds more susceptible to perceiving being phubbed as exclusionary and attributing the cause more internally, to themselves.” [36] By contrast, we found no significant differences between our American and Turkish participants. Although this may have to do with our modest sample size and lack of power, it is notable just how *similar* our findings were across the two countries, suggesting the global nature of phubbing. Even as we found no differences across country, age-related differences were significant. Younger participants (Generation Z) had higher phubbing scores when compared to older ones (Millennials). This finding is consistent with a small decrease in phubbing behavior with age based on a meta-analytic review of 79 studies [18]. Collectively, these findings suggest that age should be strongly incorporated into efforts to address phubbing. For example, with smartphone engagement and practices likely to differ significantly across age and birth cohort, approaches need to be tailored accordingly, particularly against a backdrop of exponential changes in smartphone technologies.

Neurobiological findings undergird some of our findings: First, a review of the effects of smartphones on attention suggests that smartphone use can affect people’s attention. Factors such as receiving notifications, experiencing cravings, or the fear of missing out can influence attention [37]. Furthermore, a study comparing attention test performance with and without smartphones found that the presence of smartphones was associated with lower scores in attention and processing speed [38]. These attention-related effects may explain the observed decrease in attention span among residents and patients.

Second, individuals often feel ignored or devalued due to phubbing, which can impact interpersonal relationships. Human social perception relies on the dynamic coding in the brain to detect social signals and predict emotional empathy. Moreover, resonance between individuals can enhance neural efficiency and facilitate coordination [39]. Distractibility is also associated with decreased cognitive empathy [40]. Hence, cognitive unavailability can impact relational dynamics, along with decreased empathy, consistent with findings related to phubbing. These effects can extend to both personal and professional environments, highlighting the importance of addressing digital distractions in maintaining healthy interpersonal relationships.

Third and last, a growing number of studies are examining how digital environments alter human behavior. Receiving likes or giving likes on social media triggers activation in reward-related brain regions [41], which is perceived as approval and can be rewarding. Conversely, a decrease in the number of likes received on social media posts can lead to increased post latency, ultimately influencing social media behavior [42]. Conditions such as nomophobia (fear of being without a mobile phone) and FoMO, stemming from the increased prevalence of smartphones, particularly among adolescents, can lead to problematic smartphone use or addiction [43, 44]. Since adolescence is a crucial period for brain development and adolescent brains are more sensitive to such influences, neurodevelopmental concerns may arise during this period. Indeed, a review of fMRI studies in adolescents and young adults with smartphone addiction revealed abnormalities in brain regions associated with the reward system and executive functions compared to controls [45].

Phubbing has been aptly conceptualized as “a social practice that signals relational disengagement” [46]. It can lead to disruptions in normal social interactions, to fragmented attention, and to replacement or loss of healthier activities. In the clinical environment, smartphone use and phubbing can create funneling of attention and distraction [47], and supervisors’ phubbing behavior can be mirrored by those under their guidance [48]. Because such risks can aggregate over time and result in ingrained behavioral patterns, early intervention and prevention can be critical. Despite the risks involved, smartphones are not inherently negative devices; like any tool, they can be put to the use of who wields them. Smartphones have an important role in prosocial activities, such as fostering connection between adolescents unable to drive or living at a distance from one another. In our effort to reach a more balanced utilization of these devices, we should not rush to draconian, all-or-nothing approaches. Even in the privacy of the consulting room, smartphones can be useful and legitimate tools: viewing a screen together; identifying favorite music, videos, and trends; even texting one another during a session are strategies that can prove helpful in breaking the ice, connecting, fostering rapport – in “getting it.”

Heightened self-awareness to the antecedents and consequences of phubbing can help adults take a more understanding and empathetic stance when considering how to deal with the same behavior in their children or patients. We need to find ways for those under our care—and for ourselves—to better adapt to electronic realities of our times that are here to stay, and to more effectively balance personal and professional/educational smartphone use, cognizant that smartphones can negatively impact work/school-life conflicts [49]. A self-reflective inventory of personal phubbing, along the themes we identified, can inform our guidance regarding electronic technologies for youth under our clinical care. Recognizing emotions such as boredom proneness, loneliness, or FoMO as antecedents [50], or normalization or splintered attention as consequences of phubbing, can help us better understand both ourselves and our patients. It can also help become more realistic in implementing recommendations to children, adolescents, and their families. In an effort to foster self-reflection among clinicians, we provide in **Appendix 2** illustrative vignettes of three paradigmatic phubbing scenarios (when the patient, the parent, or the clinician are the identified phubber). Clinicians can use these vignettes as a springboard for discussion, including during training or supervision.

All participants expressed the need to have better guidelines to help patients and families navigate the tricky shoals of smartphone and electronic device use. Most of them had some familiarity with one or two such guidelines, but no one felt truly confident incorporating them into practice. The most widely cited report, from the US Surgeon General [51], provides a valuable framework for appropriate social media use, with recommendations specifically tailored to policy makers, technology companies, parents and caregivers, children and adolescents, and researchers. Most proximal to the clinical domain, parents can establish tech-free zones at home to foster better in-person relationships; they can teach and model responsible online behavior; or they can report problematic content and activity. In turn, children and adolescents can limit time on platforms, block unwanted content, be careful about sharing personal information, and reach out if they need help or see harassment or abuse on the platforms. Another widely cited report from the UK’s College of Psychiatrists provides complementary guidance, including making sure that technology use becomes a core part of clinical assessment and formulation, given that “the online world can be just as important to young people as their offline world.” [20] Helpful as these recommendations are, they were not considered granular or realistic enough.

In his trade book *The Age of Anxiety* [52], Jonathan Haidt puts forth the premise that as a society we overprotect children in the real world, while we under-protect them in the virtual world – that we have failed to see and address the risks of social media on healthy development. His recommendations are clear, though perhaps aspirational at this juncture: phone-free schools; no smartphones before high school; only basic phones before roughly age 14 (with limited apps and no internet browser); and no social media before 16. He advocates for an embodied here and now, given that “in our lives with smartphones, we are forever elsewhere,” and that smartphones and other digital devices are “experience blockers” that reduce interest in non-screen-based forms of experience. Finally, Haidt makes the important point that we should not assume that disadvantaged children or those from minoritized groups have less access to the internet: they just have less protection from it. For example, families with more limited resources or lower “parental literacy” may resort to “electronic babysitting” to fill up a void or to ease challenging parent-child interactions.

Clinicians approaching phubbing and other “e-behaviors” in their young patients may have to straddle the roles of trusted therapist, guideline arbiter, and parental ally. This is no easy feat, nor one for which there is a direct formula. Two final concepts may prove helpful. First, phubbing and related behaviors have a goal to connect (to someone, to something), not just to avoid and disconnect. In some sense, they represent transitional objects or behaviors that are quite similar to or own – how many of us have not felt the “phantom” buzzing of our smartphone? How long can we go without checking our device of choice? Second, the age difference between clinicians and their patients in CAP creates a de facto generation gap between the two. This is a reality magnified by technology and its rapid advances. It is up to us to turn this potential occupational hazard into a clinical opportunity.

## Limitations

We acknowledge four main limitations, which could inform next steps: (1) Even though our sample was more than sufficient for a qualitative study [53], it did not provide sufficient power or representativeness for the quantitative component. Thus, for example, the lack of difference between the US and Türkiye may represent a Type II error; (2) We did not have an objective measure of smartphone use, which would have provided precision and a standard against which to compare self-reports; [54] (3) We did not include the perspectives of children, parents, or stakeholders to complement those of our CAP fellow participants; and (4) We did not consider clinical workload, shift schedules (if present), or electronic health records usage patterns as likely confounders.

## Conclusion

Smartphone use increased during and after the COVID-19 pandemic [55], a time in which the need for virtual connection and timely guidance accelerated reliance on and frequent use of these devices. In the wake of the pandemic, some of the benefits of the technology have diminished, making phubbing and problematic electronics use more conspicuous. As we settle back into a “post-pandemic normal,” and as we continue to be inundated with ever faster and more addictive technologies, we will continue to be sought to provide clinical guidance. In order to face this daunting challenge, we will do well by looking into our own electronic reliance and fragile social interactions.

## Supplementary Information

Below is the link to the electronic supplementary material.

Supplementary Material 1.

Supplementary Material 2.

## Data Availability

No datasets were generated or analysed during the current study.
