# Supplementary material for: When smartphones take over: a mixed methods study of phubbing in child and adolescent psychiatry
Source: Child Adolesc Psychiatry Ment Health. 2025 Aug 22;19:95. doi: 10.1186/s13034-025-00950-0 (PMC12374419; doi:10.1186/s13034-025-00950-0)
Supplement: Supplementary file 1 — Supplementary Material 1. [file 13034_2025_950_MOESM1_ESM.docx]

**APPENDIX 1**

**Interview guide**

**Beginning the conversation**

Do you know the term “phubbing”?

[After providing the standard definition…] What thoughts come to mind on hearing this?
Any resonance to yourself or others?

1. **Phubbing others**

Do you recall ever being on the phone while someone else is directly trying to interact with you in a one on one setting?

Can you describe a memorable occasion when you caught yourself phubbing others?

Can you describe a routine / unremarkable occasion when you caught yourself phubbing others? (i.e., when it had become a normalized behavior)

1. **Precursors / Precipitants**

Can you recall why? (Distracted, can’t focus, bored, urgently needed to respond to something)

Have you ever felt like you’re missing out on something when you’re away from your phone?

Have you ever tried to distance yourself from your phone?

- What do you think causes you to fail in staying away from it?
- What helps you succeed in distancing yourself from it?

1. **Who You Phub/Interpersonal Relationships**

Who do you phub?

How is your interaction with your phone when you're with friends or family?

How do you think your phone use affects your interactions with your entourage?

What about the feelings and thoughts of those phubbed

Do you feel you would be more or less likely to phub a specific person? Teacher/professor, romantic partner, authoritative figure, friend, family, etc.? Why?

1. **Smartphone Use Norms**

Whether explicitly taught to you or not, what are some norms around smartphone use during personal interactions that you tend to follow? (Ex: putting phone away/facedown at the dinner table, etc.)

Are there any norms that you feel you fail to follow?

1. **Perceptions**

Have we gained or lost more since incorporating smartphones?

Can you imagine a future time in which we gave them up?

1. **Being Phubbed By Others**

Have you personally been affected by phubbing? What emotions or messages do you feel that action communicated to you? Do you have any guesses as to why the other person phubbed you?

1. **Smartphone related questions**

Do you currently use a smartphone? If so, how frequently and for what purposes?

How long have you been using a smartphone? How old were you when you first started using a smartphone (child, adolescent, adult)?

How long can you go without your phone each day? (while awake)

How do you feel when your phone is not nearby?

Do you have any “sanctuary” hours / days when you intentionally don’t use your phone?

How would you compare the “you” on your phone and the “you” in daily life? Are there differences or similarities?

1. **Child Psychiatry and Work Related Questions**

Given your current knowledge of the field of child psychiatry and your experiences so far, what do you observe or anticipate about the usage of smartphones among children/adolescents that you work with or in general?

What is your understanding about how smartphone use affects children and adolescents?

Have you ever phubbed or been phubbed in a clinical space?

What emotions or messages do you think phubbing in an educational setting conveys to others in the room?

What basic tips do you have for parents looking for information about screen usage in children?

-How much do you incorporate these suggestions into your daily routine?

If you were unable to access your personal phone, how would your performance be affected at work?

1. **COVID-19 related questions**

In your experience, have you noticed any changes in smartphone use patterns before and after the pandemic?

If so, what are some motivations behind that change? (ex: increased use of devices including smartphones for remote education, meetings, etc.; socially distanced way to stay connected with friends and family)?

Are you more or less likely to use electronic devices including smartphones post-pandemic?

Is there anything else we did not ask that you’d like to cover?

Thank you for your time and interest!
