# Supplementary material for: When smartphones take over: a mixed methods study of phubbing in child and adolescent psychiatry
Source: Child Adolesc Psychiatry Ment Health. 2025 Aug 22;19:95. doi: 10.1186/s13034-025-00950-0 (PMC12374419; doi:10.1186/s13034-025-00950-0)
Supplement: Supplementary file 2 — Supplementary Material 2. [file 13034_2025_950_MOESM2_ESM.docx]

**APPENDIX 2**

**Illustrative Vignettes**

We provide three hypothetical scenarios in which a clinician may encounter and contend with phubbing behaviors in the practice of child and adolescent psychiatry. These scenarios have been created based on the themes, topics, and concerns described in the article. In addition to illustrating key findings, these vignettes can serve as a springboard for clinical discussion or supervision.

1. The phubbing youngster

A child presents with his father to the emergency department because the child’s behavioral issues at home have escalated dramatically. The psychiatrist arrives at the patient’s room and sees the child tapping intently at a smartphone, engrossed with an online game. Upon the psychiatrist’s entrance, the father makes several attempts to coax the child into handing over the phone to no avail. The frustrated father apologetically explains that the child was yelling and running around the waiting room. Rather than cause a scene, the father lent the child his smartphone which temporarily calmed the child. Currently, the child rebuffs any attempts to interact with the psychiatrist and yells and kicks whenever his father or the psychiatrist reaches for the phone. These events significantly delay the start of the psychiatric interview and seem to make the child less cooperative even when the phone is successfully stored away.

1. The phubbing parent
   A psychiatrist meets with a teenager and her mother for an outpatient appointment. They waited briefly prior to the psychiatrist’s entry. The psychiatrist and the teenager have built a strong rapport over a sequence of appointments, and the teenager is increasingly willing to discuss the extent of the bullying she faces at school and its impacts on her. The family discussion proceeds for the first fifteen minutes, and the patient has been tearing up upon thinking back to particularly poignant or painful moments. Partway through the meeting, the mother receives several urgent messages on her phone from her manager and becomes visibly distracted for a few minutes, during which the conversation between the patient and the psychiatrist continues. When the patient’s mother is asked a question, she looks up, apologizes, and asks for the teenager to repeat what she last said. The patient shifts uncomfortably in her seat, reluctant to describe a particularly devastating moment yet again.
2. The phubbing psychiatrist
   A child is brought to the CAP unit for an intake appointment. Thus far, the psychiatrist has managed to begin establishing rapport with the child and makes a few notes for documentation purposes on the computer at appropriate moments in the conversation. Suddenly, a handful of secure messaging notifications pop up on the psychiatrist’s computer, and she can feel the buzzing of her work phone in her pocket. The psychiatrist’s mind races as she grows worried about what the messages may imply for another of her patients. She weighs whether she should read the messages or tactfully excuse herself for a couple minutes, but ultimately consciously chooses to maintain her focus on the patient in front of her and speak to a nurse on the unit afterwards.
